# Prolonged Growth Hormone/Insulin/Insulin-like Growth Factor Nutrient Response Signaling Pathway as a Silent Killer of Stem Cells and a Culprit in Aging

**DOI:** 10.1007/s12015-017-9728-2

**Published:** 2017-02-22

**Authors:** Mariusz Z. Ratajczak, Andrzej Bartke, Zbigniew Darzynkiewicz

**Affiliations:** 1 Stem Cell Institute, James Graham Brown Cancer Center, University of Louisville, 500 South Floyd Street, Rm. 107, Louisville, KY 40202 USA; 2 Department of Regenerative Medicine, Warsaw Medical University, Warsaw, Poland; 3 Geriatrics Research, Department of Internal Medicine and Physiology, Southern Illinois University School of Medicine, Springfield, IL USA; 4 Brander Cancer Research Institute and Department of Pathology, New York Medical College, Valhalla, NY USA

**Keywords:** Longevity, VSELs, HSCs, Growth hormone, Insulin, Insulin-like growth factor, Parental imprinting, Aging, Geroprevention

## Abstract

The dream of slowing down the aging process has always inspired mankind. Since stem cells are responsible for tissue and organ rejuvenation, it is logical that we should search for encoded mechanisms affecting life span in these cells. However, in adult life the hierarchy within the stem cell compartment is still not very well defined, and evidence has accumulated that adult tissues contain rare stem cells that possess a broad trans-germ layer differentiation potential. These most-primitive stem cells—those endowed with pluripotent or multipotent differentiation ability and that give rise to other cells more restricted in differentiation, known as tissue-committed stem cells (TCSCs) - are of particular interest. In this review we present the concept supported by accumulating evidence that a population of so-called very small embryonic-like stem cells (VSELs) residing in adult tissues positively impacts the overall survival of mammals, including humans. These unique cells are prevented in vertebrates from premature depletion by decreased sensitivity to growth hormone (GH), insulin (INS), and insulin-like growth factor (IGF) signaling, due to epigenetic changes in paternally imprinted genes that regulate their resistance to these factors. In this context, we can envision nutrient response GH/INS/IGF signaling pathway as a lethal factor for these most primitive stem cells and an important culprit in aging.

## Introduction

During embryogenesis, early-development stem cells show a broad spectrum of tissue differentiation. The most primitive are totipotent stem cells, the fertilized oocyte (zygote) and the first few blastomeres in the blastula, as these cells give rise to the entire embryo proper and placenta [1]. A short time later in development the totipotency of stem cells is lost, and a population of pluripotent stem cells emerges in the inner cell mass of the blastocyst, which gives rise to all embryonic tissues except the placenta. Pluripotent stem cells are still present after blastocyst implantation in the uterus in the epiblast, from which the entire adult organism will develop. Next, among the early-development stem cells in the epiblast, some give rise to multipotent stem cells that may differentiate into cells from two different germ layers (meso-, ecto-, or endoderm) or show broad differentiation potential into cells derived from a single germ layer [2].

Further on during embryogenesis these pluripotent or multipotent stem cells became specified into tissue-committed stem cells (TCSCs), which already possess a limited ability to differentiate, being restricted to a given lineage (e.g., hematopoietic, epidermal, or neural). The proximal part of the epiblast also gives rise to primordial germ cells (PGCs), which, as precursors of gametes, carry developmental totipotency to the next generation [3]. As it will be discussed later in this review, we and others have identified a population of very small embryonic-like stem cells (VSELs) in postnatal tissues that share several characteristics with migrating PGCs [4–21]. The number of these cells in adult tissues seems to correlate positively with life span, and VSELs are proposed as a backup population for TCSCs in adult life [22–25].

One of the currently proven geropreventive measures is caloric restriction, which induces GH resistance and reduces insulin (INS) level [26–28]. Since GH stimulates liver to secrete insulin-like growth factor 1 (IGF-1), caloric restriction leads also to a decrease in the level of IGF-1 circulating in peripheral blood. Based on this, an important mechanism by which caloric restriction extends life span is the decrease in intensity of GH/INS/IGF-1 signaling [29, 30].

Since, as postulated above, VSELs are precursors of adult TCSCs and are responsible for postnatal tissue and organ rejuvenation, it is logical to assume that the robustness and overall condition of these cells affects life span. In this review we will present our working hypothesis, supported by previously generated data, that enhanced GH/INS/IGF signaling leads to premature VSEL depletion, a decrease in robustness of VSELs, and accelerates aging. Metaphorically speaking, VSELs are continuously being depleted in the “metabolic fire” generated by GH/INS/IGF signaling throughout adult life of the individual [3].

This concept has very important implications for justifying the development of new prophylactic and treatment strategies that are based on diminishing the unwanted “metabolic-side effects” of GH/INS/IGF signaling in VSELs as well as other stem cells. These strategies involve i) caloric restriction, ii) regular physical exercise, and iii) more specific drugs than those currently available (e.g., metformin, berberine, or rapamycin), which may inhibit this longevity-limiting pathway [32, 33]. We predict that the stem cell compartment targeting by pharmacotherapy to prevent premature depletion of VSELs from adult tissues will become a valuable approach to increasing human health- and life- span.

In this review we will first discuss side effects of enhanced GH/INS/IGF signaling leading to aging of somatic cells and stem cell compartment, and next we will focus on epigenetic changes in parentally imprinted genes that attenuate this signaling pathway in VSELs, protecting them from premature depletion from adult tissues.

### Aging as the Result of a Sequence of Several Adverse Molecular and Metabolic Events

Aging is an inevitable consequence of life and it has been suggested that is preprogrammed in the genes of all living organisms. It accelerates after achieving reproductive age, when the genes could be passed on to the next generation. Several mechanisms are currently proposed that accelerate this process, leading to the same result as some researchers envision of culling adult organisms that have completed the reproductive period of life.

It is well known that after cells reach the Hayflick number of divisions, their proliferative potential is exhausted, which is reflected by shortening of the tips of their chromosomes (called telomeres) [34]. The shortening of telomeres leads to telomere dysfunction, genetic aberrations and impacts cell proliferation than may end as replicative senescence. An important mechanism in the aging process is the generation of reactive oxygen species (ROS), that contribute to replication stress and oxidative DNA damage [35]. The ROS, generated in mitochondria as a product of oxidative phosphorylation, induce DNA damage including formation of the DNA double-strand breaks. The latter cause the accumulation of mutations. In this context, DNA in stem cells known to be metabolically quiescent, is more effectively protected from endogenous ROS compared to DNA in their progeny cells [36]. However, with time, even in stem cells at the reduced exposure to ROS, DNA undergoes progressive damage. Accumulation of unrepaired or incorrectly repaired DNA lesions in stem cells lowers the genome integrity leading to loss of fidelity of transcription and generation of proteins with defective function in progeny cells. The lesions at the telomeric DNA may affect cell longevity. It should be noted that hyaluronic acid, which is the major constituent of stem cell niche, by intercepting ROS protects stem cells from oxidative damage by exogenous oxidants [37]

Another important mechanism responsible for aging is impairment over time of the process of autophagy, a major degradation pathway essential for removing damaged organelles and macromolecules from the cytoplasm in eukaryotic cells, which promotes recycling of amino acids during periods of starvation [28, 38]. A decrease in autophagy activity leads to accumulation of protein aggregates, which negatively affect cell function and lead to damage and degeneration of mitochondria, thus contributing to aging [28].

In addition to the abovementioned molecular events, aging is also affected by several other biological processes, such as pathologic lipid metabolism and chronic inflammation [32, 39]. Aging is therefore a complex multigene-driven process with individual susceptibility. However, the fact that mitochondrial ROS generation contributes to aging points toward cell metabolic pathways as the basis of this process. In fact, aging is tightly connected to the intensity of metabolic impact of aerobic and anaerobic glycolysis, in which GH/INS/IGF nutrient response signaling pathway plays a crucial role [26, 28, 40–42].

### The Emerging Role of GH/INS/IGF-Regulated Pathways to Explain Aging - Lessons from Animal Models

One of the most intriguing observations related to aging is that, in all organisms, whenever there is a decrease in INS/IGF signaling (invertebrates) or GH/INS/IGF signaling (vertebrates), there is an extension of life span [40–42]. This mechanism has been observed in yeast, worms, fruit flies, and mice and also applies to humans. The genes involved in this pathway, depending on the particular species, may have different names, but all have similar effects on intracellular metabolism by targeting corresponding pathways.

The origin of this intriguing phenomenon is believed to have emerged early during evolution as it is evident by its presence in yeast. The main task of yeast, like every other organism, is to reproduce and to pass genes on to the next generation. Yeasts reproduce rapidly if there is enough carbohydrate food available in their environment. Early in their evolution, yeasts had to employ a defensive strategy when there was a food shortage - namely to slow down metabolism in order to survive until food again became available [40–42]. It has been proposed that this mechanism, developed during evolution, affected pathways involved in carbohydrate metabolism that are regulated by INS and IGF signaling and resulted in developing in vertebrates an anti-aging regulatory switch between slowing down metabolic pathways and GH/INS/IGF signaling. This evolutionarily ancient mechanism is clearly visible in addition to yeasts, for example, in other mutants of the INS/IGF pathway, and has been described in i) *Caenorhabditis elegans* (roundworm), ii) *Drosophila melanogaster* (fruit fly), and in the long living murine mutants of the GH/INS/IGF pathway [40–43]. The affected individuals are smaller in size but have an extended life span. Another recent observation from the animal world comes from Brandt’s bat, which may live up to 40 years. This bat is small (~ 4–8 g of body mass) and displays similar mutations in the GH/INS/IGF signaling pathway [44]. A similar mechanism also operates in normal individuals not affected by obvious mutations in GH/INS/IGF signaling pathways exposed for example to caloric restriction, although at a much lower level of activity.

Interesting models that support this mechanism include long-living mutant mice that have well-defined mutations in GH/INS/IGF signaling pathways. These mice are smaller in size but live much longer than their normal littermates, retaining fecundity for an extended period of life, and giving rise to viable litters even at an advanced age. These murine mutants are known in the literature as Laron, Ames, Snell, and “little” dwarf mice [24, 43].

The first strain, Laron dwarf mice are produced by targeted disruption of the GH receptor and GH binding protein encoding gene (GHR-KO or GHBP-KO mice) [43]. Despite elevated GH levels in blood, these animals do not secrete insulin-like growth factor 1 (IGF-1, also known as somatomedin C) from the liver because of a lack of functional GH receptors on hepatocytes. As a consequence of it, Laron dwarf mice have undetectable levels of IGF-1 circulating in peripheral blood, are smaller in size, but show a remarkable extension in life span and prolonged fecundity [45]. Similarly, long living are also GH releasing hormone deficient (GHRH^−/−^) mice that also have very low level of IGF-1 circulating in peripheral blood [46].

The other mutant animals, namely Ames and Snell dwarf mice, lack GH, prolactin (PRL), and thyroid-stimulating hormone (TSH) due to a defect in the “paired”-like homeodomain pituitary transcription factor Prop1 that controls development of anterior pituitary cells [47], live much longer than their normal siblings, and exhibit many symptoms of delayed aging [24]. Like Laron dwarfs, these mutants also have very low levels of circulating IGF-1 in peripheral blood. Similarly, solitary GH deficiency in “little” mice is also associated with increased life span and a decrease in IGF-1 levels circulating in peripheral blood [26].

Of importance for the topic of this review is our observation that the mentioned above long living mice during their life maintain a higher number of VSELs in bone marrow, compared to their normal littermates [24, 25].

**Fig. 1:**
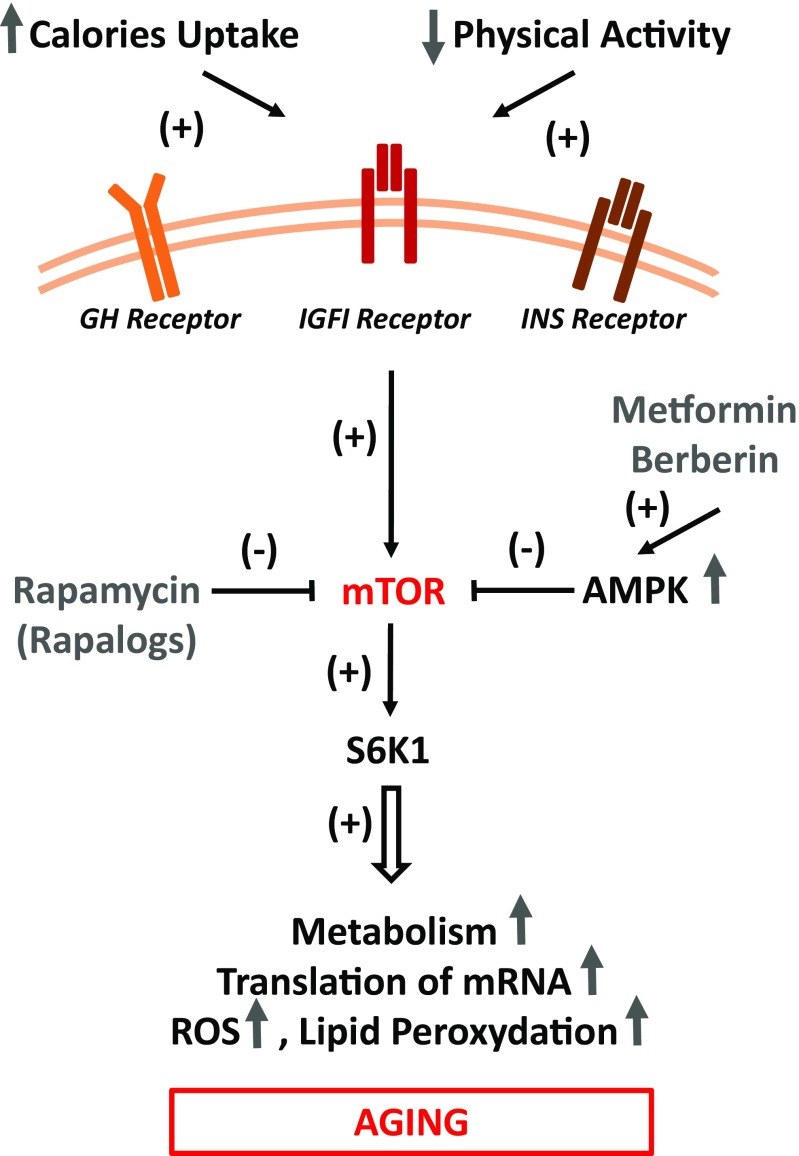
GH/INS/IGF signaling-dependent metabolic pathways that impact aging in all somatic cells and stem cells. A highly caloric diet and low levels of physical activity enhance GH/INS/IGF signaling in somatic cells in mTOR/mTORC1-dependent manner, including stem cells. The main role of mTORC1 is to activate and control translation of proteins and to exert this function TORC1 functions as a nutrient/energy/redox sensor that requires adequate energy resources, nutrient availability and oxygen abundance. However, over time, this leads to damaging, mTOR-activated intracellular processes due to e.g., ROS-mediated telomeric DNA oxidative damage, lipid peroxidation, inhibition of autophagy. The beneficial effects of AMPK activators that inhibits mTORC1 (e.g., metformin and berberine) and direct mTOR inhibitors (e.g., rapamycin) are indicated. As will be demonstrated in Fig. 2, due to epigenetic changes in methylation state of some parentally imprinted genes VSELs, similarly as PGCs are more resistant to GH/INS/IGF signaling as compared to other TCSCs and somatic cells

Another animal example is the prolonged longevity of RasGRF1-deficient and ribosomal protein S6 kinase 1 (S6 K1)-deficient mice [48–51]. Both RasGRF1 and S6 K1 are downstream signaling targets of GH/INS/IGF pathway. While RasGRF1 is a small GTP exchange factor molecule associated with the IGF-1 and INS receptors [48, 49], S6 K1 is involved in signaling from serine/threonine kinase - known as mechanistic target of rapamycin (mTOR) [51]. On the other hand, life span in wild type murine strains can be increased by pharmacological modulation of INS and IGF-1 receptor signaling with metformin [28, 32, 52] or by inhibition of mTOR, located downstream of both receptors (Fig. 1) [28, 32]. In contrast to attenuating GH/INS/IGF pathways, an increase in signaling from this axis, as seen in mice transgenic for GH or mice that are administered IGF-1 for a prolonged period, leads to accelerated aging and shortening of life span [26, 42]. In contrast these short living animals exposed permanently to high level of circulating in peripheral blood IGF-1, have as demonstrated significantly reduced number of VSELs in adult tissues [24, 25].

Based on the animal models discussed above, these studies provided important evidence for the role of the GH/INS/IGF signaling axes in regulating life span and affecting aging. The main question remains: How relevant are data obtained in mutant murine models to other larger animals? Some indirect evidence that GH/INS/IGF signaling plays a role in other species is the observation that smaller dogs have enhanced longevity compared with larger dogs [53]. In support of this correlation, the most long-living canine is the Chihuahua (~18 years, 15–25 cm tall, 2 kg body mass), and the one with shortest life span is the Irish wolfhound (~7 years, 80 cm tall, 54 kg body mass). These data, however, have to be interpreted with caution, because they may also depend on inbred defects in these animals and possibly other factors. Nevertheless, longevity has been also described in small mixed bred dogs [53]. Thus, the overall rule appears to indicate that smaller dogs live longer. The molecular mechanisms behind this intriguing fact are most likely metabolism dependent, and it would be worthwhile to study the metabolism of these animals and its relationship with the intensity of GH/INS/IGF signaling. It would be interesting to compare tissue reserve of VSELs between these animals.

As mentioned earlier, the progressive DNA damage by ROS has been considered as one of the mechanisms contributing to aging (“ROS mechanism”). However, with a few exceptions [54, 55] the evidence that antioxidants or other means of prevention of oxidative DNA damage can extend the lifespan or reduce the symptoms of aging, is scarce [56]. On other hand, as cited above, the evidence that the constitutive signaling along the axis GH/INS/IGF–mTOR–S6 K1 (“TOR mechanism”) is the driving force that accelerates aging is compelling.

It should be noted that there is an association between the ROS and mTOR mechanisms that relates to aging and DNA damage. Specifically, the persistent activation of mTOR/S6 K1 pathway is associated with translation that requires constant generation of ATP. This in turn involves enhanced oxidative phosphorylation which leads to formation of ROS and oxidative DNA damage. The damage of oncogenes or tumor suppressor genes may lead to neoplastic transformation. Consistent with this are observations that antioxidants have chemopreventive properties [57, 58], while as already mentioned, have relatively minor impact on longevity.

### Do Body Size and GF/INS/IGF Signaling Affect Life Span in Humans?

An increase in body size correlates with enhanced metabolic activity and more intense developmental engagement of the stem cell compartment, and similar studies investigating whether there is a correlation between body size and longevity have also been performed in humans [59]. Again, these reports have to be interpreted with caution, because in humans there are several genetic factors that affect average life span. Nevertheless, the available data indicate that shorter height and lower body mass correlate with prolonged life span. For example, a negative correlation between height and life span in professional baseball players in the USA has been shown as well as a negative effect of height on overall survival in the French population [59]. Likewise, there are observations that shorter American men of Japanese ancestry live longer than their taller counterparts [60]. Indirect evidence also suggests that there are low IGF-1 levels in the PB of human centenarians [61] and data that people from long living families have lower level of circulating GH [62]. In addition, there have been described functional mutations of IGF-1 receptor lowering its effectiveness in centenarians that as postulated prolongs longevity of these individuals [63].

An important and somewhat underappreciated indirect indicator of the role of IGF-1 in aging is measurement of the red blood distribution width (RDW), a component of the complete blood count indicating the size heterogeneity of the erythrocytes circulating in peripheral blood [64]. A higher RDW that is associated with a shorter life span is result of the well-known positive stimulating of IGF-1 on erythropoiesis, and indicates indirectly a role of elevated IGF-1 level in blood in senescence [65]. Based on this it has been proposed that patients with higher circulating IGF-1 levels have higher RDW and that this correlates with a decrease in life span [64].

Additional indirect evidence is the relative absence of tall individuals in populations of human centenarians [59]. Moreover, Okinawans, who are on average ~ 10–13 cm shorter than Scandinavians, have the highest proportion (500 per million) of centenarians in the world [59]. A similar finding has been reported for a population of short centenarians living on Krk Island on the Adriatic Sea [66]. It has been shown that these “little people of Krk” carry the same mutation of transcription factor Prop1 which controls development of anterior pituitary GH secreting cells as long living Ames dwarf mice [50].

Again, this evidence should be interpreted with caution, as it could be explained by several factors, such as the beneficial effects of a fish-enriched diet in Japan or of a Mediterranean diet on Krk Island. Nevertheless, these examples may also provide some hints about the role of GH/INS/IGF signaling and metabolic activity and potential impact on overall VSELs content in these individuals.

### Key Metabolic Pathways Related to Cellular Senescence and Aging

As presented above, GH/INS/IGF signaling clearly affects aging. Figure 1 shows pathways activated through the GH-, INS-, and IGF-receptors as part of a nutrient-sensing response that converges on the serine/threonine kinase mTOR - a master regulator of cell growth, metabolic function, autophagy, and metabolism - in response to nutrient-activated GH/INS/IGF signaling [28, 52]. It is well accepted that mTOR modulates the ratio between anabolic and catabolic processes in response to nutrient availability and overall cellular energy status [28, 52].

At the molecular level, mTOR functions in two distinct complexes: mTOR complex 1 (mTORC1) and mTOR complex 2 (mTORC2) [52]. mTORC1 is a protein complex composed by mTOR itself and few other regulatory proteins. Its main role is to activate and control synthesis of proteins and to exert this function TORC1 acts as a nutrient/energy/redox sensor that evaluates adequate energy resources, nutrient availability and oxygen abundance [28, 52]. mTORC1 is inhibited by rapamycin and its analogs (rapalogs), which leads to inhibition of mRNA translation and protein synthesis due to a negative effect on the two mTORC1 substrates, S6 K1 (mentioned above) and eukaryotic translation initiation factor 4E binding protein 1 (4E–BP1) [52]. This places mTOR (TORC1) complex signaling at center stage as an evolutionarily conserved regulator of life span Fig. 1 [28, 52]. In support of this role, inhibition of this regulatory complex by rapamycin or its analogs extends life span in several animal models, including yeast, round worms, fruit flies, and mice. In contrast to mTORC1, mTORC2 pathway is mainly involved in regulation of cytoskeleton, however may also affect longevity by inhibiting FOXO3a signaling [52]. However, there are some observations that TORC2 signaling may be beneficial for longevity and inhibition of TORC2 is in particular detrimental to males [67]. This opposite effects of TORC1 and TORC2 inhibition may explain in part why interventions that decrease mTOR signaling e.g., by rapamycin show greater efficacy in females [67].

As mentioned above, mice with a mutation in S6 K1 have an extended life span. Importantly, this beneficial effect of caloric restriction or S6 K1 mutation on life span can be explained by reduced mTORC1 activity downstream of GH/INS/IGF signaling (Fig. 1) [28, 52]. Moreover, both pharmacologic and genetic disruptions of this regulatory complex are sufficient to extend lifespan in several species, including mice under non-dietary restriction conditions. It has been reported that the mTORC1 complex is negatively regulated by adenosine monophosphate-activated protein kinase (AMPK), a key sensor of cellular energy status [68–70]. This kinase is an evolutionarily conserved sensor of cell metabolism and is activated by low levels of ATP. AMPK overexpression or its activation by plant-derived compounds, such as metformin, berberine, or some chemically synthesized small-molecule activators, has been reported to extend life span in experimental animal models [69, 70]. This effect is due to AMPK-mediated inhibition of GH/INS/IGF signaling in mTORC1-dependent manner (Fig. 1). Interestingly, we noticed that prolonged administration of metformin increases a number of VSELs in adult murine bone marrow.

Several clinical trials are currently being run using mTOR inhibitors, such as rapamycin or its rapalogs, as well as AMPK activators, including metformin and berberine, to extend human life span [68–71]. In order to collect definitive data, long-term studies have to be completed. It should be noted however that the direct target for metformin and berberine is respiratory complex I of electron transport chain in mitochondria. Its inhibition by either of these drugs precludes formation of ATP and thereby leads to an increase of AMP/ATP ratio. The latter provides the trigger activating AMPK which in turn inhibits mTOR signaling [72]. Considering the above mechanism these drugs, in addition to inhibiting mTOR, by preventing oxidative respiration also suppress formation of ROS. Interestingly, we noticed that prolonged administration of metformin increases a number of VSELs in adult murine bone marrow.

### The Unexpected Role of Class III Histone Deacetylases (Sirtuins) and their Role in Prolonging Life Span

Histone deacetylases (HDACs) are enzymes that remove acetyl groups on histones, which allows these proteins to wrap DNA around core histones of nucleosome more tightly [73]. HDACs also exert other pleiotropic effects in cells by interacting with intracellular targets. The most intriguing among the HDACs are the class III enzymes, which in mammals consist of seven members (SIRT-1–7) that emerged during evolution from the yeast *Sir2* gene [73].

Of the seven mammalian sirtuins, SIRT-1 is the closest homolog of yeast Sir2 and is the most-studied mammalian sirtuin. SIRT-1 predominantly localizes to the cell nucleus and shows several pleiotropic effects beside its role in histone deacetylation [74–76]. Specifically, it may deacetylate p53 and peroxisome proliferator-activated receptor gamma coactivator 1-alpha (PGC1α) and thus inhibit apoptosis and enhance mitochondrial function and biogenesis, respectively. Moreover, the SIRT-1-regulated acetylation state of FOXO transcription factors is thought to selectively direct these factors to certain targets in the cell and to regulate cell metabolism and stress responses [74, 75]. Other newly identified novel functions of SIRT-1 include i) neuroprotection, ii) liver regeneration, and iii) delayed replicative senescence of fibroblasts [74].

There are several studies demonstrating a positive effect by SIRT-1 in prolonging longevity, which can be explained in the context of GH/INS/IGF signaling and caloric restriction. SIRT-1 is reportedly stimulated by resveratrol, although the reality of this latter effect is still under debate [74]. What is highly relevant for the topic of this review, SIRT-1 also chaperones a de novo methyltransferase known as DNMT3L [76–78], a mechanism that maintains quiescent state of VSELs and prevents their proliferation [22]. We noticed that inhibition of SIRT-1 by nicotinamide or valporic acid leads to increase in proliferation of VSELs both in vivo and in vitro cultures [22]. Interestingly, an inhibition of SIRT-1 by nicotinamide or valporic acid has been recently postulated to play an important role in promoting efficient expansion of human LT-HSCs [79–83]. This may be related as we will discuss below to expansion of VSELs, that could be specified into long term repopulating hematopoietic stem cells (LT-HSCs) [83, 84].

### Effects of Changes in GH/INS/IGF Signaling on the Stem Cell Compartment

While all these discussed above effects of GH/INS/IGF signaling apply to all somatic cells, at the same time they are also highly relevant for stem cells as well.

To assess the effect of GH/INS/IGF signaling on stem cells, we evaluated the hematopoietic stem cell (HSC) compartment in Laron and Ames dwarf mice and observed that these mice, with undetectable plasma levels of circulating IGF-1, have an enhanced number of LT-HSCs and hematopoietic progenitors compared with control littermates [24, 25]. By contrast, the number of HSCs was reduced in GH transgenic mice, which have enhanced GH/INS/IGF signaling due to high levels of circulating IGF-1 in peripheral blood [24]. Our observations were recently confirmed in an elegant study by another group [85]. We also reported that prolonged caloric restriction and physical activity enhances the number of HSCs in wild type mice [86, 87]. This effect is again most likely related to attenuation of GH/INS/IGF signaling [29, 30].

In our studies, an increase in the number of HSCs in i) Laron and Ames dwarf animals, ii) wild type mice under prolonged caloric restriction, and iii) mice subjected to regular daily physical activity correlated with an increase in the number of VSELs [86, 87]. This is highly important, because, as we and others have demonstrated, VSELs are precursors of long term repopulating HSCs (LT-HSCs) [83, 84]. Interestingly, other investigators have also reported that prolonged caloric restriction had a positive effect on the number of skeletal muscle stem cells [88] and that physical activity increases the number of neural stem cells in the brain [89]. The potential involvement of VSELs in these latter phenomena requires further studies.

The adult stem cell compartment has also been evaluated in other experimental models of murine longevity, and these results corroborate the concept that augumented GH/INS/IGF signaling has a negative effect on these cells. For example, enhanced hematopoietic potential and LT-HSC activity have been reported in mentioned above mouse S6 K1 mutants [51], which display defective signaling downstream from the mTORC1 (Fig. 1). Moreover, in vivo administration of an mTORC1 inhibitor, rapamycin, leads to rejuvenation of HSCs and intestinal stem cell functions in older animals [90–92]. Unpublished results from our group revealed as mentioned above an increase in the number of VSELs and HSCs in mice treated for a prolonged period of time with metformin, which, negatively affects mTORC1 via AMPK and additionally activates SIRT-1.

The most intriguing results, however, are from animals with manipulated sirtuin expression [93, 94]. Specifically, while upregulation of SIRT1 by small-molecule activators (SRT1720 or SRT3025) had a beneficial effect on extending life span and expanding HSCs in wild type mice [91, 92], mice with inducible hematopoietic SIRT-1 knockout displayed accelerated hematopoietic aging due to an accelerated decrease in the number of HSCs [93, 94]. These results indicate that SIRT-1 is a guardian of HSCs during life. Similar results were recently observed in SIRT-3-KO and SIRT-7-KO animals [95, 96], and it would be interesting to evaluate quantity of VSELs in tissues of these animals.

### Aging, the Stem Cell Compartment, and GH/INS/IGF Signaling from the Perspective of very Small Embryonic-like Stem Cells (VSELs) Residing in Adult Tissues

As mentioned above, evidence has accumulated for the scenario that during embryogenesis stem cells related to epiblast stem cells or migrating PGCs escape specification into TCSCs. Instead, they retain pluripotent character and survive as a population of VSELs into adulthood, forming a reserve pool of precursors for TCSCs in adult issues [22, 97–99].

These small cells are slightly smaller than mature erythrocytes and have been purified by multiparameter flow cell sorting from adult tissues, including bone marrow, umbilical cord blood, and mobilized peripheral blood, and are very well characterized at the molecular level [97–99]. In addition to hematopoietic tissues they are also detected in adult organs, including gonads, brain, liver, heart, and skeletal muscles [100]. The small size of these cells (~3–5 μm in mice and 4–7 μm in humans) and the paucity of mitochondria are signs of their quiescence and low metabolic activity [97–99]. BM-isolated VSELs have been shown to remain as precursors of TCSCs for several types of cells, including hematopoietic cells, mesenchymal cells, endothelial cells, lung alveolar epithelial cells, and cardiomyocytes [11–18, 83, 84]. At the same time, VSELs isolated from murine and human gonads have been proposed to be precursors of male and female gametes [6, 8–10].

Murine and human BM-derived VSELs: i) are very rare (~0.01–0.001% of nucleated BM cells); ii) express several pluripotent stem cell markers, including Oct4, Nanog, Rex-1, and SSEA-1 (murine VSELs) or SSEA-4 (human VSELs); iii) contain sparse, round mitochondria; and iv) have large nuclei filled with unorganized euchromatin [4, 5]. Evidence from our and other groups indicates that VSELs are a population of migratory cells, and their number increases in peripheral blood during stress situations related to tissue or organ injuries [22]. Therefore, besides being a backup population for TCSCs in adult life, VSELs may play a physiologically important surveillance role in repairing certain minor tissue injuries [22, 23].

**Fig. 2:**
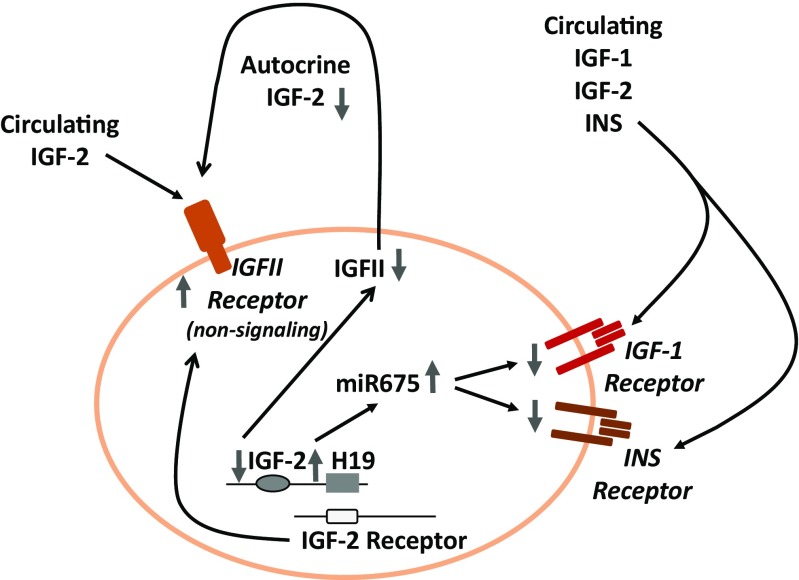
Changes in the methylation state of parentally imprinted genes lead to attenuation of GH/INS/IGF signaling in VSELs. VSELs are deposited in adult tissues as a backup population for tissue-committed stem cells (TCSCs). Due to erasure of paternal imprinting at the *Igf2-H19* locus, VSELs do not express endogenous IGF-2 and, through the activity of H19 gene-derived miRNA675, downregulate expression of the IGF-1 and INS receptors, which decreases their sensitivity to the circulating IGF-1, INS and IGF-2 activating GH/INS/IGF signaling axis. At the same time, due to hypermethylation of the DMR at the *Igf2R* locus by upregulating expression of the non-signaling IGF-2 receptor (which serves as molecular bin for IGF-2), these cells additionally attenuate responsiveness to circulating IGF-2. During aging, gradual hypermethylation at the *Igf2-H19* locus is observed, which leads to an increase in expression of autocrine IGF-2 and a decrease in H19-expressed miR675, which leads to an increase in expression of the IGF-1 and INS receptors. This results in age-related increased sensitivity to GH/INS/IGF signaling and age-mediated VSEL depletion. As a consequence, there is a decrease in VSEL-generated TCSCs, which impairs tissue and organ rejuvenation. Moreover, VSELs deposited in adult tissues may, over time, become more quickly depleted by chronically elevated circulating levels of IGF-1 and INS, which engage the IGF1R and INSR expressed by these cells. This mechanism may contribute to accelerated aging observed in situations with high circulating levels of IGF-1 and INS (e.g., high calorie uptake)

What is most relevant to the topic of this review, the highly quiescent state of VSELs in adult tissues is regulated by epigenetic changes in certain paternally imprinted genes that are involved in GH/INS/IGF signaling (Fig. 2). Overall, epigenetically regulated parental genomic imprinting is an important mechanism that ensures the parent-of-origin-specific monoallelic transcription of parentally imprinted genes (depending on whether the gene is from the maternally or paternally inherited chromosome) and plays a crucial role in embryogenesis and the pluripotency of early-development stem cells, including VSELs [97]. The expression of parentally imprinted genes is regulated by DNA methylation at differentially methylated regions (DMRs), which are CpG-rich *cis-*regulatory elements within a particular parental gene locus [101].

It has been demonstrated that VSELs residing in adult tissues erase some of the paternally methylated imprints (e.g., at the mouse *Igf2–H19* and *Rasgrf1* loci); however, at the same time they hypermethylate some of the maternally methylated imprints (e.g., at the mouse locus encoding the Igf2 receptor [*Igf2R*] and at the mouse *Kcnq1-p57* ^*KIP2*^ and *Peg1* loci) [97]. As a result of these epigenetic changes in the methylation state of DMRs in paternally imprinted genes, VSELs highly express growth-repressive genes (*H19, p57* ^*KIP2*^ *,* and *Igf2R*) and at the same time downregulate growth-promoting genes (*Igf2* and *Rasgrf1*) [97]. What is important for topic of this review, several of these genes are involved in GH/INS/IGF signaling [31, 97].

Of particular interest is the *Igf2-H19* locus, which encodes insulin-like growth factor 2 (IGF-2), and this protein ligand signals through the IGF-1 and INS receptors [101]. The same locus also transcribes the non-coding RNA H19, which gives rise to several miRNAs, including miR675-3p and miR675-5p, and these downregulate expression of the respective IGF-1 and INS receptors on VSELs (Fig. 2) [31, 97]. Another gene locus affected by epigenetic erasure of DMRs is *Rasgrf1,* which encodes a small GTP exchange factor involved in signaling from the IGF-1 and INS receptors [31, 48, 49]. Moreover, epigenetic changes in VSELs due to hypermethylation at the maternally imprinted *Igf2-R* locus also lead to upregulation of the IGF-2 receptor, which is not a signaling receptor and serves as a “molecular bin” to prevent interaction of IGF-2 with the IGF-1 and INS receptors [31].

Therefore, epigenetic reprograming changes observed in VSELs lead to a decrease in GH/INS/IGF signaling in these cells, keeping them in a quiescent state and preventing their premature depletion in adult tissues [31]. These epigenetic changes may be additionally enhanced by caloric restriction and regular physical activity [85–89] as well as by administration of certain drugs, including metformin, berberine, or rapamycin [68–71]. This quiescent state of VSELs is also most likely promoted by other activators of AMPK as well as of SIRT-1 [74, 75].

Interestingly, as mentioned above an inhibition of SIRT-1 by nicotinamide or valporic acid has been recently postulated to play an important role in promoting an efficient expansion of human LT-HSCs [79–82]. Since SIRT-1 is a chaperone of DNMT3L [77, 78], and DNMT3L is required for re-methylation of erased regulatory loci at parentally imprinted genes including *Igf2-H19* [31], we postulate that expansion of LT-HSCs in presence of nicotinamide or valporic during SIRT-1 inhibition occurs from expanded VSELs.

This differentiation of VSELs into LT-HSCs, as we have proposed, is fostered by re-methylation of erased loci in parentally imprinted genes that leads e.g., to increase in expression of IGF2 and downregulation of H19 [31]. Our data was recently confirmed in an elegant in vivo murine model by independent group of investigators who demonstrated that maternal type of methylation state - erasure of imprinting at *Igf2-H19* loci regulates quiescence of LT-HSCs [102].

We propose that modulation of VSEL robustness in various adult tissues is crucial for therapeutic strategies to prolong life span. VSEL robustness also explains at the stem cell level the role of GH/INS/IGF signaling in aging. Based on this reasoning, VSELs are at center stage as a crucial target for better understanding the effects of different strategies attenuating GH/INS/IGF signaling in promoting longevity [103]. These strategies include caloric restriction, physical activity, and the effect of drugs that attenuate GH/INS/IGF signaling [68–71, 85–89]. A decline in the number of VSELs residing in adult tissues as result of an increase in GH/INS/IGF signaling, e.g., due to a high caloric diet or a low level of physical activity, results in accelerated aging. An important gatekeeper to prevent premature depletion of VSELs and to keep them quiescent is SIRT-1. This may explain beneficial effects of metformin, berberine and rapamycin as drugs that promote longevity. On other hand by inhibiting SIRT-1 using valporic acid or nicotinamide we were recently able to force VSELs to proliferate and expand ex vivo for potential therapeutic purposes in chemically defined in vitro cultures [22].

Nevertheless, this epigenetic modulation of expression of genes involved in GH/INS/IGF signaling that protects VSELs from premature depletion from adult tissues is attenuated as demonstrated in mice with age due to gradual methylation of erased DMRs at *Igf2-H19* and *RasGrf1* loci [24]. This mechanism contributes to age-dependent depletion of VSELs, and as we envision contributes to aging.

## Conclusions

We have presented evidence indicating that strong GH/INS/IGF signaling has an accelerating effect on aging and leads to a decrease in stem cell number including VSELs. Therefore, by targeting GH/INS/IGF signaling using highly specific inhibitors, we may be able to develop new, potent, and side effect-free therapeutic strategies that could fulfill the dream of an “ambrosia” or “fountain of youth” to prolong human both life span and health span. The strategies may also involve regenerative medicine employing VSELs harvested at young age or individual’s cells harvested, genetically reprogrammed, expanded in vitro, and used for autologous transplant. One possible approach would be to harvest stem cells from the umbilical blood of the newborn, store it cryogenically and use when the donor reaches an old age in hope of “rejuvenation” of at least some his/her organs or functions.
